# A distinct histopathological variant of a malignant melanoma with perivascular pseudorosettes: A case report

**DOI:** 10.3892/ol.2013.1430

**Published:** 2013-06-28

**Authors:** MITSUAKI ISHIDA, MUNEO IWAI, KEIKO YOSHIDA, AKIKO KAGOTANI, HIDETOSHI OKABE

**Affiliations:** Department of Clinical Laboratory Medicine, Division of Diagnostic Pathology, Shiga University of Medical Science, Otsu, Shiga 520-2192, Japan

**Keywords:** malignant melanoma, perivascular pseudorosette, rosettes

## Abstract

Although a rare condition, rosette formation in malignant melanoma has been previously documented. The present study describes the second documented case of malignant melanoma with perivascular pseudorosettes. A 38-year-old male presented with a black nodule on his back. Histopathological study revealed diffuse proliferation of neoplastic cells in the dermis and subcutis. A section of the tumor (~30%) was composed of a conventional malignant melanoma component. The remaining area was comprised of medium-sized polygonal cells with slightly eosinophilic cytoplasm and small-to-medium, round nuclei. Melanin pigment was rarely observed. A noteworthy observation was the presence of perivascular pseudorosette formations, which were characterized by their radial arrangement around the blood vessels, with a perivascular, anuclear zone. Immunohistochemically, the neoplastic cells were diffusely positive for S-100 protein and Melan-A and focally positive for HMB-45. Clinicopathological analyses of cases of malignant melanoma with rosette formations revealed that the types of rosette included the Homer-Wright type (two cases), perivascular pseudorosettes (two cases) and an unclassifiable type (one case). Immunohistochemical analysis is a useful method for forming a diagnosis as Melan-A or HMB-45 are generally expressed in all cases. Rosette formation in malignant melanoma is a distinct histopathological variant and may be an under-recognized phenomenon. Therefore, its recognition is significant for obtaining an accurate diagnosis of malignant melanoma.

## Introduction

Malignant melanoma occasionally exhibits a variety of cytomorphological and architectural features and includes the balloon cell, rhabdoid, small cell, signet-ring cell, myxoid and adenoid (pseudoglandular) types (1). Although rare, rosette formation has previously been documented in malignant melanoma and Spitz nevus (1–5). The present study describes the second documented case of malignant melanoma with perivascular pseudorosettes and provides a review of the clinicopathological features of malignant melanoma with rosette formation. Written informed consent was obtained from the patient.

## Patient and methods

### Case Report

A 38-year-old male with no past history of malignant melanoma presented with a gradually enlarging nodule on his back. A physical examination revealed that the nodule was black and measured 35×30 mm in diameter. The left axillary lymph nodes were enlarged. Computed tomography demonstrated multiple nodules in the liver, which were clinically suspected to be metastatic lesions. A total resection of the back nodule and the left axillary lymph nodes was performed.

### Materials

The formalin-fixed, paraffin-embedded tissue blocks of the resected skin specimen and lymph nodes were cut into 3-μm thick sections, deparaffinized and rehydrated. Each section was stained with hematoxylin and eosin and used for immunostaining. The immunohistochemical analyses were performed using an autostainer (XT system Benchmark; Ventana Medical System, Tucson, AZ, USA) according to the manufacturer’s instructions. The following primary antibodies were used: A mouse monoclonal antibody against CD56 (CD564; Novocastra Laboratories, Ltd., Newcastle upon Tyne, UK), a mouse monoclonal antibody against chromogranin A (DAK-A3; DAKO Cytomation, Glostrup, Denmark), a mouse monoclonal antibody against glial fibrillary acid protein (GFAP; 6F2; DAKO), a mouse monoclonal antibody against HMB-45 (HMB-45; Novocastra), a mouse monoclonal antibody against Melan-A (A103; Novocastra), a rabbit polyclonal antibody against S-100 protein (Nichirei Bioscience, Tokyo, Japan) and a mouse monoclonal antibody against synaptophysin (27G12; Novocastra).

## Results

**Figure 1:**
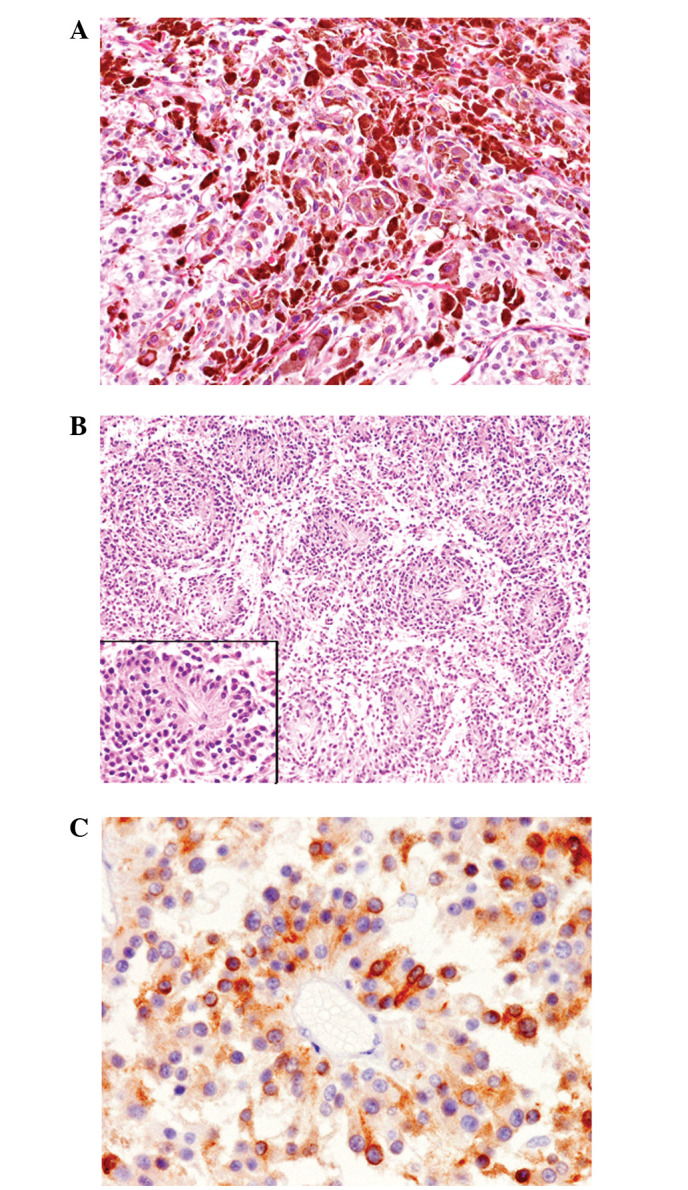
Histopathological and immunohistochemical features of the back nodule. (A) The conventional component of the malignant melanoma is composed of large polygonal cells with large round nuclei. The majority of the tumor cells contain melanin pigment (hematoxylin and eosin staining; magnification, ×200). (B) Perivascular pseudorosettes. Medium-sized polygonal cells with round nuclei and slightly eosinophilic cytoplasms. (Inset) These tumor cells are arranged radially around blood vessels with a perivascular anuclear zone (hematoxylin and eosin staining; magnification, ×100; inset, ×400). (C) Melan-A is expressed in the tumor cells that form the perivascular pseudorosettes (x400).

The histopathological study of the resected back nodule revealed a diffuse proliferation of neoplastic cells accompanied by scattered geographical necrotic foci from the entire dermis to the subcutis. The upper section of the lesion (~30% of the tumor) was composed of a conventional malignant melanoma component. The section was comprised of large polygonal neoplastic cells that contained large nuclei with or without conspicuous nucleoli. The majority of these cells contained the melanin pigment within the cytoplasm (Fig. 1A). The remaining area of the tumor was composed of medium-sized polygonal cells with slightly eosinophilic cytoplasm and small-to-medium round nuclei with or without nucleoli. Melanin pigment was rarely observed in these neoplastic cells. A noteworthy observation was the presence of perivascular pseudorosette formations within these areas (Fig. 1B). These tumor cells were arranged radially around the blood vessels, with a perivascular anuclear zone (Fig. 1B, inset). Mitotic figures were scattered in the entire lesion (17/10 high-power fields).

Immunohistochemical studies revealed that S-100 protein and Melan-A were diffusely expressed in the two components (Fig. 1C). HMB-45 was diffusely positive in the conventional component, but focally expressed in the areas with perivascular pseudorosettes. Synaptophysin, chromogranin A, CD56, and GFAP were not expressed in the two components.

The left axillary lymph nodes contained metastatic malignant melanoma with the two components and their immunohistochemical characteristics were the same as those of the back lesion.

According to these findings, a diagnosis of malignant melanoma with perivascular pseudorosettes accompanied by lymph node metastases was made.

## Discussion

In diagnostic pathology, rosettes are classically referred to as discrete cell clusters showing peripheral nuclear palisading and a central space. Although rosettes are commonly considered to be of neuroepithelial differentiation, they are occasionally observed in other types of tumors, including neuroendocrine carcinomas, granulosa cell tumors and thymomas. Certain histopathological subtypes of rosettes are well known, including the Homer-Wright type and perivascular pseudorosettes. Homer-Wright rosettes are characterized by a radial arrangement of cells with centrally situated fibrillary material and are often observed in neuroblastomas, medulloblastomas and primitive neuroectodermal tumors. Perivascular pseudorosettes are composed of columnar cells arranged radially around the blood vessels, with a perivascular anuclear zone. This structure is a well-known characteristic feature of an ependymoma (2–4).

Although rare, rosette formation has been previously documented in malignant melanomas (1–4). Table I summarizes the clinicopathological features of the four previously reported cases of malignant melanoma with rosette formation, in addition to the present case. The types of rosettes include the Homer-Wright type (two cases), perivascular pseudorosettes (two cases) and an unclassifiable type (one case). Banerjee and Harris described a case of metastatic small-cell melanoma in the bone marrow that was accompanied by perivascular pseudorosettes (1), and the present case is the second documented case of this type. In the two cases, the rosettes were present at the primary site and at the metastatic lymph nodes. In the other two cases, the rosettes were observed only at the metastatic sites. Although the case described by Alonso *et al* described melanin pigment within the cytoplasm of the neoplastic melanocytes forming the Homer-Wright rosette, melanin pigment was barely observed within the cytoplasm of the rosette-forming tumor cells in the present case and in the case reported by Falconieri *et al*(4). Therefore, differential diagnoses from other types of rosette-forming tumors, including neuroblastomas and malignant peripheral nerve sheath tumors, are necessary. However, Melan-A or HMB-45 are expressed in all cases of malignant melanoma with rosette formation (Table I). Therefore, immunohistochemical analyses may facilitate the achievement of a correct diagnosis.

Although the mechanism of rosette formation in malignant melanoma remains unclear, it has been speculated that the neoplastic melanoma cells share the same capabilities for differentiation as neural crest precursors (4).

In conclusion, the present study describes the second documented case of a malignant melanoma with perivascular pseudorosettes. Rosette formation in malignant melanoma is a distinct histopathological variant and may be an under-recognized phenomenon. Additional studies are required to clarify the clinicopathological features of this variant of melanoma, whose recognition is significant for obtaining an accurate diagnosis of a malignant melanoma.

## Figures and Tables

**Table I:** Clinicopathological features of malignant melanoma with rosettes.

First author/s, year (ref.)	Case no.	Age, years	Gender	Location	Type of rosette	Immunohistochemistry
Banerjee and Harris, 2002 (1)	1	N/A	N/A	Bone marrow	Perivascular	N/A
Pföhler *et al, 2003*(2)	2	29	Female	Upper arm/lymph node	Unclassifiable	S-100 protein^+^, HMB-45^+^
Alonso *et al*, 2003 (3)	3	61	Male	Lymph node	Homer-Wright	S-100 protein^+^, Melan-A^+^, chromogranin A^−^, synaptophysin^−^, neurofilament^−^, GFAP^−^
Falconieri *et al*, 2010 (4)	4	43	Female	Back	Homer-Wright	S-100 protein^+^, Melan-A^+^, HMB-45^+^
Present Case	-	38	Male	Back/lymph node	Perivascular	S-100 protein^+^, Melan-A^+^, HMB-45^+^ (focally), chromogranin A^−^, synaptophysin^−^, neurofilament^−^, GFAP^−^

N/A, not available; GFAP, glial fibrillary acid protein.
